# Prediction and Analysis of SARS-CoV-2-Targeting MicroRNA in Human Lung Epithelium

**DOI:** 10.3390/genes11091002

**Published:** 2020-08-26

**Authors:** Jonathan Tak-Sum Chow, Leonardo Salmena

**Affiliations:** 1 Department of Pharmacology and Toxicology, University of Toronto, Toronto, ON M5S 1A8, Canada; jonathants.chow@mail.utoronto.ca; 2 Princess Margaret Cancer Centre, University Health Network, Toronto, ON M5G 2C4, Canada

**Keywords:** microRNA, SARS-CoV-2, coronavirus, lung epithelia, cellular antiviral defence

## Abstract

Severe acute respiratory syndrome coronavirus 2 (SARS-CoV-2), an RNA virus, is responsible for the coronavirus disease 2019 (COVID-19) pandemic of 2020. Experimental evidence suggests that microRNA can mediate an intracellular defence mechanism against some RNA viruses. The purpose of this study was to identify microRNA with predicted binding sites in the SARS-CoV-2 genome, compare these to their microRNA expression profiles in lung epithelial tissue and make inference towards possible roles for microRNA in mitigating coronavirus infection. We hypothesize that high expression of specific coronavirus-targeting microRNA in lung epithelia may protect against infection and viral propagation, conversely, low expression may confer susceptibility to infection. We have identified 128 human microRNA with potential to target the SARS-CoV-2 genome, most of which have very low expression in lung epithelia. Six of these 128 microRNA are differentially expressed upon in vitro infection of SARS-CoV-2. Additionally, 28 microRNA also target the SARS-CoV genome while 23 microRNA target the MERS-CoV genome. We also found that a number of microRNA are commonly identified in two other studies. Further research into identifying bona fide coronavirus targeting microRNA will be useful in understanding the importance of microRNA as a cellular defence mechanism against pathogenic coronavirus infections.

## 1. Introduction

On March 11 2020, the World Health Organization (WHO) declared the outbreak due to the novel coronavirus, severe acute respiratory syndrome coronavirus 2 (SARS-CoV-2), a pandemic. SARS-CoV-2 is the virus that causes the coronavirus disease (COVID-19), which is characterized by severe respiratory illness [1] and cardiovascular disease [2]. As of August 9, 2020, there are 19,462,112 cases worldwide and 722,285 deaths (mortality rate of ~3.7%) confirmed by the WHO. Since the declaration of the pandemic, there has been an enormous effort to develop vaccines and treatments. Many antiviral drugs approved for other viral infections are under investigation for repurposing for COVID-19, including hydroxychloroquine and remdesivir [3]. Antibody-based treatments are also being investigated as potential drug candidates [3,4]. Additionally, there are numerous clinical trials in progress for potential vaccines of various platforms, ranging from DNA vaccines to inactivated virus vaccines [4,5]. With no signs of the virus slowing to date, there is an urgent need to develop vaccines, novel drug therapies and new strategies to combat this and future pandemics due to coronaviruses. A question that remains unanswered regarding COVID-19 is why some people have severe symptoms while others do not. Therefore, knowledge of an individual’s susceptibility to SARS-CoV-2 infection and other viral insults through identifying specific critical biomarkers may guide future antiviral prevention and treatment strategies.

MicroRNA (miRNA) are a class of small RNA molecules that function to suppress gene expression post-transcriptionally [6]. Genes encoding miRNA are transcribed to generate unprocessed RNA transcripts called pri-miRNA, which are further processed into pre-miRNA by the nuclear microprocessor complexes composed of the ribonuclease Drosha and the RNA-binding protein DGCR8 (also known as Pasha). The resulting single stranded pre-miRNA are then transported into the cytoplasm to undergo further processing by Dicer into a duplex miRNA. Unwinding of the duplex into a mature 19 to 25 nucleotide 5’-miRNA and 3’-miRNA is mediated by the miRNA-induced silencing (miRISC) complex composed of Argonaute (Ago) and GW182 families of proteins [7,8]. A mature miRNA can suppress the expression of various classes of RNA transcripts by guiding the miRISC to a sequence on an RNA transcript called a miRNA response element (MRE) to induce either RNA degradation or translation repression [9,10]. miRNA binding typically involves perfect complementarity between the MRE and a sequence of six to eight bases at the 5’ end of the mature miRNA, known as the miRNA seed [11,12]. In addition to complementarity with the seed region, miRNA must share at least partial complementarity with the MRE in the 3’ region of the mature miRNA sequence [12]. Due to the overall partial complementarity of binding to a MRE, a single miRNA may bind to multiple MREs on diverse RNA transcripts [11,12,13].

miRNA are best known as regulators of endogenous RNA transcript stability and translation [9,10]. Importantly, miRNA have also been reported to serve as an intracellular cellular defence mechanism, which can curtail viral infection by directing miRISC to viral genomic RNA [14]. For instance, hsa-miR-196, hsa-miR-296, hsa-miR-351, hsa-miR-431, and hsa-miR-448 were observed to attenuate hepatitis C (HCV) viral replication in vitro. This group of miRNA were also found to be induced by interferon-β (IFNβ) treatment, a standard treatment regimen for HCV-infected patients [15]. This finding highlights an intriguing cross-talk between the immune system through release of immune cytokines such as interferon and the deployment of miRNAs to combat viral infections [15]. Another recent study used a high-throughput reporter screen of miRNA from human and mouse respiratory epithelial cells to identify hsa-miR-127-3p, hsa-miR-486-5p, and hsa-miR-593-5p as contributors to the antiviral defence against influenza A virus by targeting the genomes of the H3N2 and attenuated PR8 (H1N1) viral strains [16]. Additionally, hsa-miR-1-3p was found to contribute to this antiviral defence mechanism by targeting ATP6V1A, a host supportive factor for influenza A replication [16]. Another study identified hsa-miR-324-5p as a suppressor of the highly pathogenic influenza A virus by targeting both the viral genome of the H5N1 strain and the host CUEDC2 gene, which is a negative regulator of the antiviral interferon pathway [17]. Finally, expression profiling of influenza-A infected cells identified 20 miRNA and 1286 mRNA that were differentially expressed; among these differentially expressed genes, 107 miRNA-mRNA interactions were correlated with antiviral defence in these cells [18].

Given the wealth of evidence supporting a role for miRNA in host cell antiviral defence mechanisms, we sought to identify human miRNA that have the potential to target the SARS-CoV-2 genome. Our analyses identified several miRNA with predicted MREs in the SARS-CoV-2 genome. Furthermore, we assessed the expression levels of these miRNA candidates in lung epithelial tissue and SARS-CoV-2 infected cells [19]. Additionally, we cross-referenced the miRNA we identified in this study with those identified in other reports [20,21] to generate a list of higher confidence miRNA predicted to target the SARS-CoV-2 genome. Altogether these data have allowed us to make inference towards susceptibility of infection and possible endogenous miRNA-mediated protective mechanisms.

## 2. Materials and Methods

### 2.1. TargetScan (v7.2)

TargetScan (http://www.targetscan.org/vert_72) is a web-based miRNA target prediction algorithm that predicts miRNA targets in whole genomes of various species by searching for the presence of sequences in each genome that match seed regions annotated in their database of seed regions. TargetScan defines a seed region to be positions 2 to 7 (from the 5’ end) of a mature miRNA. From their latest release (v7.2), we obtained an annotated list of mature miRNA with the corresponding seed regions and miRNA family.

### 2.2. RNA22 (v2)

RNA22 (https://cm.jefferson.edu/rna22/Interactive/) is a web-based miRNA target prediction tool with a downloadable version for remote use. This interactive tool allows for MRE prediction in various species, and in custom sequences. This prediction tool identifies MREs in a given target sequence based using a computational pattern-based methodology [22]. In addition to identifying MREs, RNA22 also predicts the heteroduplex formed by the miRNA and the target RNA sequence and calculates the free energy of this heteroduplex [22]. We used this prediction tool to identify MREs in the SARS-CoV, MERS-CoV, and SARS-CoV-2 viral genomes. Only significant miRNA-MRE predictions (*p* < 0.05) were considered for subsequent analyses.

### 2.3. TCGA-LUAD Dataset

We obtained the entire miRNA-Seq expression dataset from the TCGA-LUAD project and utilized only the 46 matched “normal” tissue specimens for our analyses. This dataset is available from the TCGA Research Network database: https://www.cancer.gov/tcga.

### 2.4. Viral Genome Analysis

We obtained the reference genomes for SARS-CoV, MERS-CoV, and SARS-CoV-2 from GenBank (https://www.ncbi.nlm.nih.gov/genbank/). The following accession numbers were used as search queries for each genome: NC_004718.3 (SARS-CoV), NC_019843.3 (MERS-CoV), and NC_045512.2 (SARS-CoV-2).

### 2.5. miRNA Differential Expression Analysis

miRNA-sequencing data (GEO accession: GSE148729 [19]) were accessed from the NCBI GEO database [23]. In this study, we only considered read count data from SARS-CoV-2 and mock infected Calu3 cells 24 h post-infection [19]. The edgeR software package available in R [24] was used to calculate the differential expression of miRNA in SARS-CoV-2 vs. mock infected Calu3 cells, and perform multidimensional scaling analysis of the GSE148729 dataset.

## 3. Results

### 3.1. Target Prediction in SARS-CoV-2

**Figure 1:**
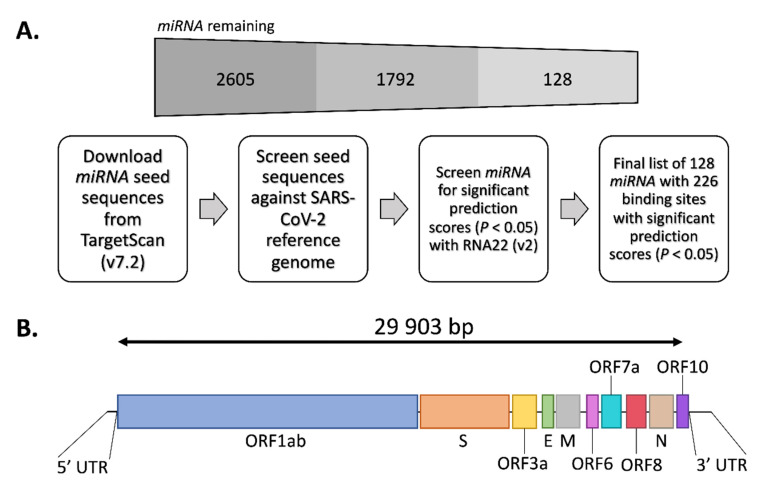
Computational identification of microRNA (miRNA) with predicted miRNA response elements (MREs) in the severe acute respiratory syndrome coronavirus 2 (SARS-CoV-2) reference genome. (**A**) Computational pipeline used to identify the 128 candidate miRNA with at least one predicted MRE. The remaining number of miRNA remaining after each step is shown above. (**B**) Schematic of the SARS-CoV-2 reference genome (NC_045512.2) with key features shown.

Using miRNA databases, target prediction tools and a computational pipeline (outlined in Figure 1A), we sought to identify miRNA with potential to target the SARS-CoV-2 RNA genome. For this we first accessed all known human miRNA seed sequences from the latest release of TargetScan (v7.2) [11]. Searches for the presence of miRNA seed-matches in the SARS-CoV-2 reference genome (NC_045512.2; Figure 1B) resulted in 1792 candidates. As miRNA binding to target RNA transcripts is promiscuous [12], we further assessed the binding strength and significance of the 1792 candidate miRNA to the SARS-CoV-2 genome using the RNA22 (v2) target prediction tool [22]. This analysis resulted in the identification of 128 miRNA that had predicted MREs with a statistically significant RNA22 prediction score (*p* < 0.05). The 128 miRNA were predicted to have a total of 226 MREs in SARS-CoV-2 (Table 1.)

### 3.2. Candidate miRNA Expression in Normal Lung Epithelia

**Figure 2:**
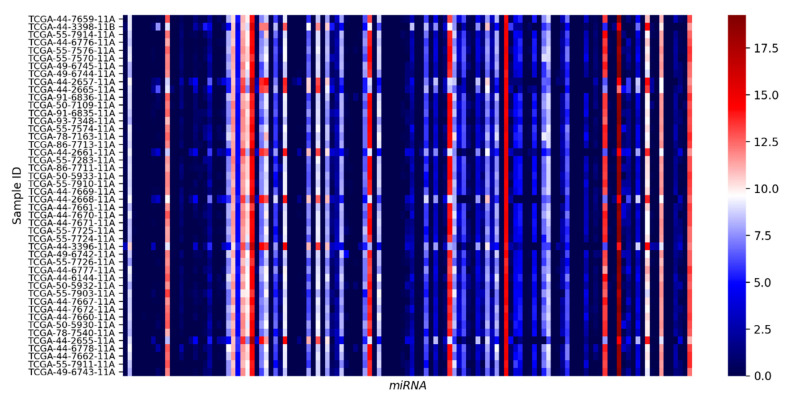
Analysis of miRNA expression (log2 transformed) from 46 normal lung tissue control samples in the TGCA-LUAD dataset.

To gain insight into the baseline levels of candidate miRNA in human lung epithelia, we curated miRNA expression data from 46 “normal” lung tissue specimens, which serve as control baseline samples in the TCGA-LUAD dataset [25]. In this investigation, we observed that miRNA expression was quite consistent between different patients. The most highly expressed miRNA included hsa-mir-143, hsa-let-7a-1, hsa-let-7b, hsa-let-7f-2, hsa-mir-101-1, hsa-mir-103a-2, hsa-mir-182, hsa-let-7c, hsa-mir-151a, hsa-let-7e, and hsa-mir-145 using a cut-off of log2 transformed expression of 10. Notably, a large majority of the 128 candidate miRNA have very low to no expression in the lung epithelia (Figure 2).

### 3.3. miRNA Expression Changes upon SARS-CoV-2 Infection

**Figure 3:**
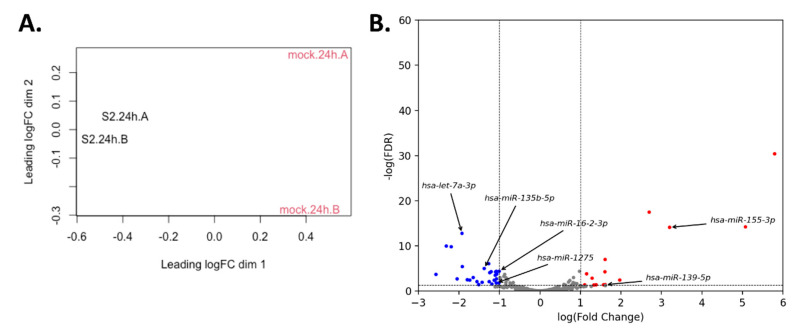
Differential miRNA expression analysis of Calu3 cells infected with SARS-CoV-2 or mock 24 h post-infection from GSE148729. (**A**) Multidimensional scaling analysis of samples and replicates from GSE148729. Samples infected with mock are in red and samples infected with SARS-CoV-2 are in black. (**B**) Significantly differentially expressed miRNA. A miRNA was considered differentially expressed if it had log(fold-change) magnitude > 1 and FDR < 0.05. Significantly downregulated miRNA are in blue and significantly upregulated miRNA are in red.

Changes in host miRNA expression levels upon viral infection is well-documented. To understand how SARS-CoV-2 infection can alter the expression of miRNA in lung epithelial cells, we conducted differential expression analysis (DEA) of miRNA-sequencing data derived from SARS-CoV-2-infected Calu3 cells [19]. Multidimensional scaling analysis demonstrates clear clustering of SARS-CoV-2-infected samples and control samples indicating that SARS-CoV-2 infection can indeed alter miRNA expression patterns (Figure 3A). DEA identified 45 miRNA that were differentially expressed (FDR < 0.05; | log(fold-change) | > 1), of which 17 miRNA were upregulated and 28 miRNA were downregulated (Figure 3B and Table 2). When cross-referenced with our 128 miRNA candidates, we observed that six candidate miRNA were both differentially expressed and had a significant miRNA prediction score (Figure 3B). Four were observed to be downregulated (hsa-let-7a-3p, hsa-miR-135b-5p, hsa-miR-16-2-3p, and hsa-miR1275), whereas two were upregulated (hsa-miR-155-3p and hsa-miR-139-5p).

### 3.4. Conservation of miRNA Binding in Coronaviruses

**Figure 4:**
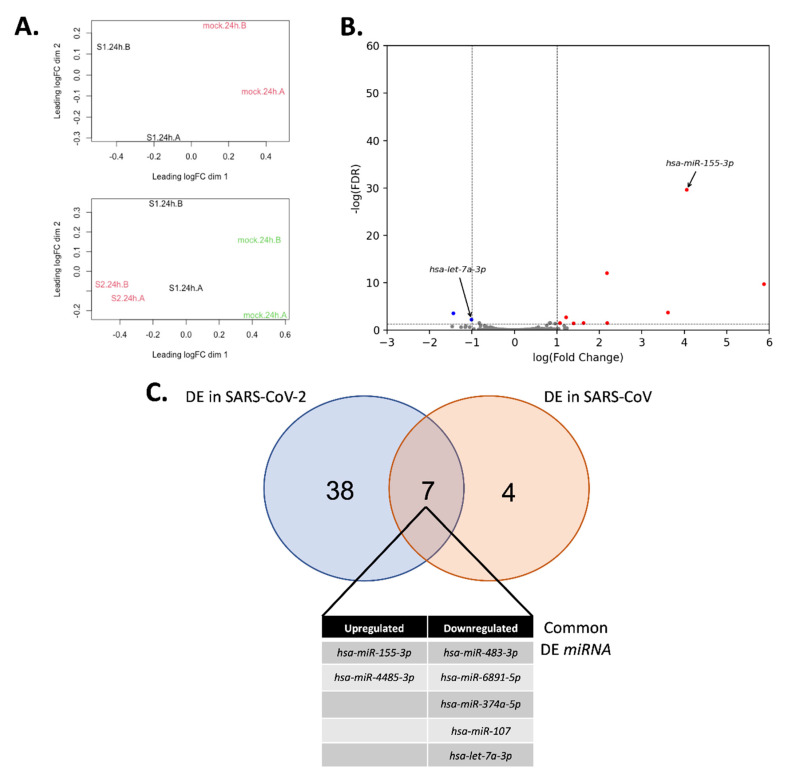
Differential miRNA expression analysis of Calu3 cells infected with SARS-CoV or mock 24 h post-infection from GSE148729. (**A**) Multidimensional scaling analysis between SARS-CoV and mock infected cells (top) and between SARS-CoV-2, SARS-CoV and mock infected cells. Samples infected with mock are in green, samples infected with SARS-CoV-2 are in red and samples infected with SARS-CoV are in black. (**B**) Significantly differentially expressed miRNA. Significantly downregulated miRNA are in blue and significantly upregulated miRNA are in red. miRNA were considered differentially expressed if the |log(fold-change)| > 1 and FDR < 0.05. (**C**) Commonly differentially expressed miRNA between SARS-CoV-2 and SARS-CoV infection.

Since SARS-CoV-2 is among a handful of coronaviruses that are pathogenic to humans, we aimed to assess if any of the 128 miRNA had conserved or non-conserved binding sites in the genomes of other prominent coronaviruses. We focused on the SARS-CoV (NC_004718.3) and MERS-CoV (NC_019843.3) reference genomes as these coronaviruses can cause severe respiratory disease and have previously caused notable outbreaks worldwide [26,27,28]. Notably, the SARS-CoV-2 genome was only ~79% and ~50% similar to the SARS-CoV and MERS-CoV genomes, respectively [29]. Using the RNA22 (v2) algorithm to find predicted binding sites, we found that only 28/128 and 23/128 had a predicted binding site (*p* < 0.05) in the SARS-CoV or MERS-CoV genomes, respectively (Table 3). Notably, none of these binding sites are conserved in either of the SARS-CoV or MERS-CoV genomes despite the high homology that exists between these viruses and SARS-CoV-2. The dataset generated by Wyler et al. (2020) also contains miRNA-sequencing data for SARS-CoV infected Calu3 cells 24 h post-infection; similar to SARS-CoV-2, multidimensional scaling analysis revealed very distinct miRNA expression profiles between SARS-CoV and mock infected cells (Figure 4A, left). Furthermore, multidimensional scaling of the miRNA-sequencing from SARS-CoV-2, SARS-CoV and mock infected Calu3 cells demonstrates that each sample type produces distinct clusters indicating each infection produces different changes in miRNA expression patterns (Figure 4A, right). DEA of Calu3 cells infected with SARS-CoV revealed that only hsa-miR-155-3p (upregulated) and hsa-let-7a-3p (downregulated) out of the 128 miRNA we identified in this study, were differentially expressed (Figure 4B). Comparing the differentially expressed miRNA in SARS-CoV-2 and SARS-CoV infected Calu3 cells, only seven miRNA were commonly upregulated whereas only two miRNA were commonly downregulated (Figure 4C).

### 3.5. Comparison to Other miRNA SARS-CoV-2 Studies

**Figure 5:**
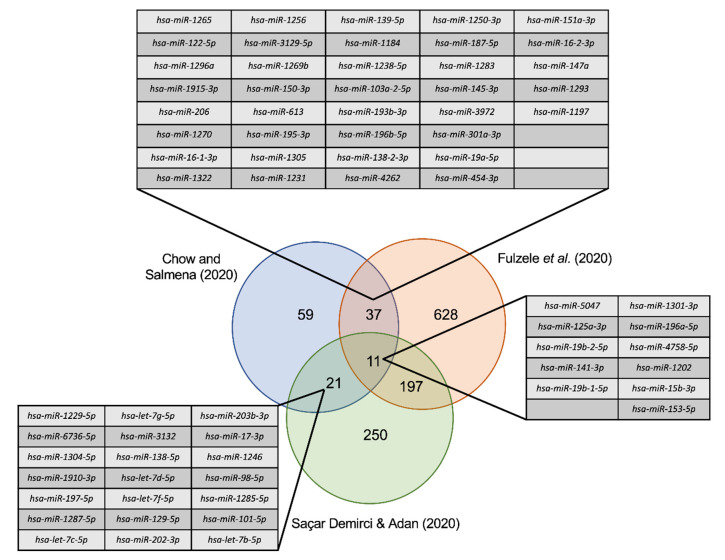
Commonly identified miRNA with other computational prediction studies.

To date, several reports have identified miRNA predicted to have binding sites in the SARS-CoV-2 genome using different target prediction algorithms. Identifying commonly predicted miRNA from our analysis and these other reports will provide greater confidence in these candidates given that different methodologies were utilized. Upon comparison of our list of 128 miRNA candidates with miRNA identified in previous reports revealed several miRNA in common. Specifically, there were 48 miRNA in common with Fulzele et al. (2020) [20], 32 miRNA in common with Saçar Demirci and Adan (2020) [21], and 11 miRNA in common between all three studies (Figure 5). The 11 common miRNA include hsa-miR-5047 hsa-miR-1301-3p, hsa-miR-125a-3p, hsa-miR-196a-5p, hsa-miR-19b-2-5p, hsa-miR-4758-5p, hsa-miR-141-3p, hsa-miR-1202, hsa-miR-19b-1-5p, hsa-miR-15b-3p and hsa-miR-153-5p.

## 4. Discussion

In this study we utilized a miRNA discovery pipeline to identify 128 putative miRNA with MREs in the SARS-CoV-2 genome. Given the number of reports that point to a role for miRNA as part of a cellular defense mechanism to mitigate infection by RNA viruses, we hypothesized that high expression of miRNA within our set of 128 candidates will provide protection against SARS-CoV-2 infection. By cross-referencing our list of 128 candidate miRNA against other publicly available miRNA expression databases we aimed to gain insight into features of these miRNA as they relate to infections by coronaviruses such as SARS-CoV-2.

Firstly, by utilizing miRNA expression profiles from normal tissue controls from the TCGA-LUAD project dataset, we observed that only a small number of our miRNA candidates (11/128) have high expression in normal lung epithelia. Unfortunately, these 11 were not further validated as good candidates by further analyses. Notably, the large majority of candidates are expressed at very low levels in normal lung tissues. We posit that low expression of SARS-CoV-2-targeting miRNA may underscore a lack of natural endogenous protection against infection of the lung epithelium. It has also been proposed that the selective tissue tropism of some viruses may be due to the tissue specific expression of miRNA [30]. As viruses have evolved, selective pressure will have undoubtedly removed antiviral miRNA binding sites from the RNA genome. However, some of these antiviral binding sites may be preserved due to mutual exclusion of the virus and the tissue specificity expression of miRNA corresponding to these sites [30]. This may be the case with the miRNA and their predicted binding sites in the SARS-CoV-2 genome that we have identified here in our analyses as many of these miRNA have very low expression in the lung tissue.

Secondary structure is also known to affect miRNA target binding [31,32,33]. Although evidence from the literature only indicates secondary structures in the 5’ and 3’ untranslated regions in related coronaviruses (SARS-CoV and MERS-CoV) [34], it is likely that SARS-CoV-2 has evolved to also adapt complex secondary structure in its coding regions to evade endogenous RNA interference (RNAi) attack. However, it is known that coronaviruses undergo RNA-dependent RNA synthesis mediated by an RNA-dependent RNA polymerase (RdRp) as a key step in their life cycle, producing full length genomic RNA and shorter subgenomic RNA [35]. Given that these are de novo products, it is likely that they do not adapt these secondary structures as they are produced given that the RdRp is required to have access to the RNA template. It is then conceivable that endogenous host miRNA could mediate an RNAi-mediated attack on these de novo products. However, further research will be required to investigate the ability for endogenous miRNA to target either the viral genome or de novo viral genomic and subgenomic RNA.

We next explored the possibility that SARS-CoV-2-targeting miRNA could be upregulated upon SARS-CoV-2-infection, and as a consequence function as an induced antiviral protective mechanism. For this we analysed data from a recent study that performed miRNA-sequencing on SARS-CoV-2-infected and mock-infected Calu3 cells in vitro [19]. We conducted a DEA and cross-referenced differentially expressed miRNA with our candidate SARS-CoV-2-targeting miRNA. We observed that six out of 128 candidate miRNA were differentially expressed in vitro—only two of which were upregulated—indicating that SARS-CoV-2-targeting miRNA are likely not substantially induced upon infection. The near absence of activation of SARS-CoV-2-targeting miRNA indicates that lung epithelia may have a low capacity to mount any miRNA-mediated defence against SARS-CoV-2. Nevertheless, a number of miRNA are differentially expressed suggesting that miRNA may be part of a response to infection. Future studies assessing differential miRNA expression from SARS-CoV-2 infected lung epithelia patients’ samples may provide further insight into intracellular protective and other responsive mechanisms.

In the last two months, a number of other studies have reported host miRNA with predicted binding sites in the SARS-CoV-2 genome. Fulzele et al. (2020) recently identified 873 miRNA with predicted MREs in 29 SARS-CoV-2 patient samples [20]. Interestingly, 48 miRNA from Fulzele et al. (2020) study were also present in our list of 128 miRNA candidates. Similarly, Saçar Demirci and Adan (2020) identified 479 miRNA with a predicted MREs in the SARS-CoV-2 genome [21] and there are 32 overlapping miRNA with our list of 128 miRNA. Among these three studies 11 miRNA were found to be common. Given that the three analyses were performed independently using different prediction pipelines, overlapping hits may provide higher confidence candidate SARS-CoV-2-targeting miRNA.

miRNA with bona-fide MREs in the SARS-CoV-2 genome with low expression in patient lung epithelia could potentially be administered exogenously as synthetic miRNA-mimic drugs (hereafter, agomirs). Treatment with formulations of individual or cocktails of agomirs may have a role in the prevention or frontline treatment of patients with coronavirus infection and severe acute respiratory disease with a goal of preventing further viral transmission. While there are no agomir-based drugs currently approved for coronavirus infections, several studies have reported positive results for this drug class in the in vivo protection against influenza A (H1N1) [16] and hepatitis B [36] viral infections. With the current rate of infection worldwide, drugs that can limit or prevent the transmission of coronavirus infections may fill an unmet global need. Further investigation on validating the ability for miRNA to target the SARS-CoV-2 genome will be required prior to the development of any agmoir-based therapies, however, our results provide a basis for this research.

The current pandemic has clearly demonstrated that viral infections can pose a major threat to human health worldwide. The RNA genome and the mechanisms of infection whereby coronavirus and other viruses expose their genome provide an opportunity for endogenous miRNA attack and therapeutic targeting with agomirs. Thus, identifying miRNA that target coronaviruses gives a better understanding of the changes in miRNA expression in patients upon infection and may provide further critical insights into miRNA-associated protective mechanisms and possible therapeutic strategies. For instance, in addition to miRNA targeting and destroying a viral genome directly, it is also reported that a virus can highjack endogenous miRNA function for its own purposes including binding to cellular targets that are crucial to propagate viral life cycles and the course of infection as demonstrated by the observation that hepatitis C virus (HCV) replication is dependent on a liver-specific miR-122 [37]. Indeed, inhibition of miR-122 by antagomirs can reduce viral titers in HCV infected patients. DNA viruses can harbour endogenous miRNA in their genomes [37], and there is an increasing amount of evidence suggesting that retroviruses can also harbour miRNA [38]. In addition, it has been reported that some RNA viruses such as influenza A can produce small viral RNA (svRNA) from its genome, independent of host miRNA biogenesis machinery [39]. This has also been reported for the SARS-CoV genome where three svRNA were identified, one of which was suggested to contribute to the pathology in the lungs [40]. Given the conservation between SARS-CoV and SARS-CoV-2, the production of svRNA is possible but yet to be reported. Finally, the role of endogenous host miRNA in the antiviral response is highly debated as there are contrasting bodies of literature [30,41,42,43].

## 5. Conclusions

In this study, we suggest that the low expression and lack of differential expression of miRNA predicted to target the SARS-CoV-2 genome may in part underlie the lack of a miRNA-associated protective mechanism, and thereby promote susceptibility of the lung epithelia to infection. Increasing the expression of these miRNA (either endogenously or therapeutically) in respiratory epithelial cells may provide a cellular defense against viral infection and propagation. As such, further research into identifying bona fide miRNA that can target viral genomes may be useful in designing novel agomir-based therapies to heighten the protective capacity of cells against pathogenic coronavirus infections.

## Figures and Tables

**Table 1:** miRNA with a significant predicted binding site (*p* < 0.05) in the SARS-CoV-2 reference genome (NC_045512.2).

miRNA with a Significant Predicted Binding Site				
hsa-let-7i-5p	hsa-miR-182-3p	hsa-let-7d-5p	hsa-miR-19b-2-5p	hsa-miR-142-3p.1
hsa-miR-4701-3p	hsa-miR-1270	hsa-miR-1184	hsa-miR-138-1-3p	hsa-miR-1284
hsa-miR-1273g-5p	hsa-miR-4298	hsa-miR-150-3p	hsa-miR-202-3p	hsa-miR-138-2-3p
hsa-miR-1265	hsa-miR-16-1-3p	hsa-miR-15b-3p	hsa-miR-103a-2-5p	hsa-miR-1208
hsa-miR-6736-5p	hsa-let-7b-5p	hsa-let-7f-5p	hsa-miR-1185-2-3p	hsa-miR-1273g-3p
hsa-miR-122-5p	hsa-miR-197-5p	hsa-miR-129-5p	hsa-miR-1976	hsa-miR-4262
hsa-miR-1229-5p	hsa-miR-1322	hsa-miR-1202	hsa-miR-301a-3p	hsa-miR-1273e
hsa-miR-6511a-5p	hsa-miR-1275	hsa-miR-4665-5p	hsa-miR-206	hsa-miR-17-3p
hsa-miR-1269a	hsa-miR-196a-5p	hsa-miR-1267	hsa-miR-98-5p	hsa-miR-1304-3p
hsa-miR-4420	hsa-miR-1305	hsa-miR-1231	hsa-miR-4500	hsa-miR-19a-5p
hsa-miR-1301-3p	hsa-miR-152-5p	hsa-miR-1238-5p	hsa-miR-147a	hsa-miR-1302
hsa-miR-1256	hsa-miR-3132	hsa-miR-19b-1-5p	hsa-miR-3116	hsa-miR-1237-3p
hsa-miR-1910-3p	hsa-miR-138-5p	hsa-miR-3118	hsa-miR-141-3p	hsa-miR-191-3p
hsa-miR-1915-5p	hsa-miR-134-5p	hsa-miR-6874-3p	hsa-miR-185-3p	hsa-miR-1299
hsa-miR-1292-5p	hsa-miR-1298-3p	hsa-miR-1199-5p	hsa-miR-134-3p	hsa-miR-146a-3p
hsa-let-7g-5p	hsa-miR-195-3p	hsa-miR-1254	hsa-miR-1238-3p	hsa-miR-613
hsa-miR-3129-5p	hsa-miR-1250-5p	hsa-miR-1224-5p	hsa-miR-193b-5p	hsa-let-7f-2-3p
hsa-miR-1287-5p	hsa-miR-1269b	hsa-miR-550a-3p	hsa-miR-1250-3p	hsa-miR-196b-5p
hsa-let-7c-5p	hsa-miR-153-5p	hsa-miR-202-5p	hsa-miR-142-3p.2	hsa-miR-3972
hsa-let-7e-5p	hsa-miR-125a-3p	hsa-miR-187-5p	hsa-miR-135a-5p	hsa-miR-143-5p
hsa-miR-1304-5p	hsa-miR-139-5p	hsa-miR-101-5p	hsa-miR-1251-3p	hsa-miR-151a-3p
hsa-miR-4758-5p	hsa-miR-5047	hsa-miR-1972	hsa-miR-1185-1-3p	hsa-miR-203a-3p.2
hsa-miR-135b-5p	hsa-miR-155-3p	hsa-miR-145-3p	hsa-miR-1233-3p	hsa-miR-1293
hsa-miR-203b-3p	hsa-miR-4458	hsa-miR-454-3p	hsa-miR-4518	hsa-miR-1291
hsa-let-7a-3p	hsa-miR-16-2-3p	hsa-miR-193b-3p	hsa-miR-1283	hsa-miR-1285-5p
hsa-miR-1246	hsa-miR-1197	hsa-miR-124-5p		

**Table 2:** Significantly up and downregulated miRNA in Calu3 cells infected with SARS-CoV-2 or mock from GSE148729.

Differentially Expressed miRNA			
Upregulated miRNA		Downregulated miRNA	
*hsa-miR-4485-3p*	*hsa-miR-501-5p*	*hsa-let-7a-3p*	*hsa-miR-26b-3p*
*hsa-miR-483-3p*	*hsa-miR-181-5p*	*hsa-miR-374a-5p*	*hsa-miR-23c*
*hsa-miR-6891-5p*	*hsa-miR-4745-3p*	*hsa-miR-374a-3p*	*hsa-miR-374c-5p*
*hsa-miR-4284*		*hsa-miR-194-5p*	*hsa-miR-374b-3p*
*hsa-miR-4463*		*hsa-miR-4454*	*hsa-miR-26a-1-3p*
*hsa-miR-155-3p*		*hsa-miR-135b-5p*	*hsa-miR-365a-3p*
*hsa-miR-483-5p*		*hsa-miR-16-2-3p*	*hsa-miR-365b-3p*
*hsa-miR-12136*		*hsa-miR-23b-3p*	*hsa-miR-181-3p*
*hsa-miR-155-5p*		*hsa-miR-21-5p*	*hsa-miR-940*
*hsa-miR-107*		*hsa-let-7f-1-3p*	*hsa-miR-362-3p*
*hsa-miR-125b-5p*		*hsa-miR-429*	*hsa-miR-1275*
*hsa-miR-29b-2-5p*		*hsa-miR-5701*	*hsa-miR-1296-5p*
*hsa-miR-139-5p*		*hsa-miR-450b-5p*	*hsa-miR-126-5p*
*hsa-miR-299-5p*		*hsa-miR-7-1-3p*	*hsa-miR-548d-3p*

**Table 3:** miRNA with a significant predicted binding site in the SARS-CoV-2 reference genome that also target either the SARS-CoV (NC_004718.3) or MERS-CoV (NC_019843.3) reference genomes.

SARS-CoV		MERS-CoV	
*hsa-let-7i-5p*	*hsa-miR-1208*	*hsa-let-7i-5p*	*hsa-let-7a-2-3p*
*hsa-let-7b-5p*	*hsa-miR-4500*	*hsa-let-7c-5p*	*hsa-miR-103a-2-5p*
*hsa-let-7c-5p*	*hsa-miR-101-3p.2*	*hsa-let-7e-5p*	*hsa-miR-4500*
*hsa-let-7e-5p*	*hsa-let-7a-2-3p*	*hsa-let-7g-5p*	*hsa-miR-10a-5p*
*hsa-let-7g-5p*	*hsa-miR-98-5p*	*hsa-let-7b-5p*	*hsa-miR-101-5p*
*hsa-miR-1202*	*hsa-let-7i-3p*	*hsa-miR-1202*	*hsa-miR-10b-5p*
*hsa-miR-105-3p*	*hsa-let-7g-3p*	*hsa-let-7d-5p*	*hsa-miR-1185-2-3p*
*hsa-miR-1224-5p*	*hsa-miR-1197*	*hsa-let-7f-5p*	*hsa-miR-103b*
*hsa-miR-4458*	*hsa-miR-101-3p.1*	*hsa-miR-98-5p*	*hsa-miR-1185-1-3p*
*hsa-let-7f-5p*	*hsa-miR-1199-5p*	*hsa-miR-1224-5p*	
*hsa-miR-1205*	*hsa-miR-103b*	*hsa-miR-1184*	
*hsa-miR-1184*	*hsa-miR-10b-5p*	*hsa-miR-105-3p*	
*hsa-miR-1183*	*hsa-miR-1178-3p*	*hsa-miR-4458*	
*hsa-miR-103a-2-5p*	*hsa-let-7d-5p*	*hsa-miR-1183*	
